# Dietary intake of Senegalese adults

**DOI:** 10.1186/1475-2891-9-7

**Published:** 2010-02-18

**Authors:** Cheryl AM Anderson, Scarlett Bellamy, Mindy Figures, Charnita Zeigler-Johnson, Mohamed Jalloh, Elaine Spangler, Margerie Coomes, Serigne Gueye, Timothy R Rebbeck

**Affiliations:** 1 Department of Epidemiology, Johns Hopkins University Bloomberg School of Public Health, Welch Center for Prevention, Epidemiology and Clinical Research, (2024 E. Monument Street, Ste 2-600), Baltimore, MD (21205), USA; 2 Department of Biostatistics and Epidemiology, University of Pennsylvania, School of Medicine, Center for Clinical Epidemiology and Biostatistics, (423 Guardian Drive), Philadelphia, PA (19104), USA; 3 University Cheikh Ante Diop Dakar, Hôpital Général de Grand Yoff, Dakar, Senegal

## Abstract

The aim of this work is to identify major food sources and dietary constituents of Senegalese adults. We conducted a cross-sectional study, using a single 24-hour dietary recall interview. Foods were classified into food groups based on similarities in nutrient content or use. Food groups included foods consumed individually, or as part of food mixtures such as stews, soups, or sandwiches. Median consumption (amount/day) of each food was determined and examined by relevant subgroups. Participants were 50 healthy Senegalese men, aged 20-62 years recruited at the Hôpital Général de Grand Yoff in Dakar, Senegal and from Sendou village, a rural area outside Dakar. A total of 90 foods and beverages were identified and classified into 11 groups. Sixty-five percent of foods identified could be classified as meats, grains, or fruits/vegetables. Fruits and vegetables comprised 42% (38/90) of all foods; meats 12% (11/90); and grains 11% (10/90). Sauces (6%, 5/90), sweets (4%, 4/90), and desserts (4%, 4/90) were also reported. The most common fruits/vegetables reported were potato, carrot, mango, and lettuce; commonly reported grains were bread and rice; and commonly reported meats were fish, beef, and ox. There were no differences in reported daily intake of each food by age, ethnicity, education, or residence. Most foods reported were traditional to the Senegalese diet, despite the increasing availability of Western foods in Senegal.

## Findings

Diet-related diseases and conditions such as certain cancers, cardiovascular disease, hypertension, and diabetes are prevalent worldwide. Research on the role of diet in disease prevention is strongly linked to the conduct of precise dietary assessment in populations. Critical elements of dietary assessment include knowledge of the type, frequency, and amount of foods consumed; as well as the expected diversity in dietary intake patterns of a study population. Given the high prevalence of diet related disorders in populations, dietary assessment efforts are needed for the conduct of nutritional epidemiological studies and for treating diet related diseases and conditions. In Senegal, little is known about the modern diet of adults as most studies of nutrition in this population have focused on malnutrition and growth retardation in childhood [1-6].

The lack of nutritional epidemiological studies in Senegal is related to lack of standardized dietary assessment tools. The creation of these tools may have been hindered by a hypothesized low variability in dietary intake for foods or food groups that are associated with chronic diseases. However, there are no empirical data to support this notion. In fact, it is possible that there has been an epidemiologic transition [7] and an accompanying nutrition transition [8] resulting from an increase in non-traditional lifestyles and a concomitant increase in intake of non-traditional foods by Senegalese. This would result in greater heterogeneity of intake in the modern diet, and it has been predicted that a dramatic shift in causes of mortality from nutrition-related chronic diseases will occur in developing countries, including the Sub-Sahara [9]. Therefore, the primary objective of this study was to describe the Senegalese diet. Although we are unable to examine diet-disease relationships in this study, this work will serve as a prelude to a more formal examination of dietary influences on the health of Senegalese.

Participants were healthy, Senegalese men aged 20-62 years (n = 50) who were recruited at the Hôpital Général de Grand Yoff (but were not hospitalized) in Dakar, Senegal (n = 40) and from Sendou village - a neighboring rural area (n = 10). In general, a convenience sample was used and recruitment was conducted by a combination of physician referral and word of mouth. All study procedures were approved by the University of Pennsylvania Institutional Review Board and by the Commission Ethique et Evaluation (IRB) at Hôpital Général de Grand Yoff under FWA 00002772.

Participants agreed to in-person interviews and signed an informed consent form. Food intake data were collected by a trained interviewer during a single 24-hour dietary recall interview. Interviews were conducted with the assistance of a native Senegalese physician and translator. As is typical in the 24-hour recall interview, participants were asked to remember and report all food and beverage items consumed in the previous 24 hours. The interviewer probed in detail about each food and beverage item reported. Participants were asked details about the name, place and time of consumption of the meal, method of preparation, amount consumed, condiments added, recipes, brand names or name of menu items for restaurants, take-out foods, or other foods eaten outside of the home. During the interview, participants were shown visual aids to assist them in estimating amount consumed. Demographic information such as age, tribal affiliation, education, residence, marital status, as well as health behaviors, medical history, and family history were also collected.

There were two primary analytic goals: 1) Identify and classify foods consumed into broad categories. Categories were primarily defined by traditional Western food groups; and 2) descriptively summarize the amount consumed of the most frequently reported foods. Frequencies and percentages were calculated in order to determine the most popular foods consumed. The median and interquartile ranges (IQR) for daily intake are presented for the top 25% of food items reported in each food group. For example, if there were 11 foods reported in a food group, the median daily consumption for the top 3 foods was presented. We compared median daily consumption by age, residence, and tribal affiliation using non-parametric Wilcoxon tests. All analyses were performed using SAS version 9.1 or higher.

Characteristics of study participants are shown in Table 1. Mean age was 31 years, body mass index was 22.7 kg/m^2^, and the men represented various ethnic/tribal backgrounds. Most (64%) were married and had at least some college education (40%).

**Table 1:** Characteristics (mean and range) of study participants

Variable	Mean (range)
Age (yrs)	31.0 (20.0, 62.0)

Body Mass Index (kg/m^2^)	22.7 (17.8, 31.3)

	**Freq (%)**

Prepare own meals	3 (6)

Live in urban area	40 (80)

Married	32 (64)

Ever smoked	16 (32)

Education	
Primary/illiterate (includes Coranic School education)	14 (28)
First half of secondary school	5 (10)
Last half of secondary school	11 (22)
College education	20 (40)

Ethnicity (Maternal)	
Arab	1 (2)
Diolas	4 (8)
Mandingues	6 (12)
Mencayne	1 (2)
Pulaar	12 (24)
Sereer	11 (22)
Soninke	1 (2)
Wolof	14 (28)

Ethnicity (Paternal)	
Arab	1 (2)
Diolas	4 (8)
Mandingues	4 (8)
Mencayne	1 (2)
Pulaar	13 (26)
Sereer	10 (20)
Soninke	1 (2)
Wolof	16 (32)

There were 90 unique foods and beverages reported by study participants (Table 2). These foods could be classified into 11 distinct categories: meat and meat alternatives; milk/dairy products; grains; fruits and fruit juices; vegetables and vegetable juices; sweets/sweeteners; sauces; fats; condiments/spices; desserts; and beverages (not fruit/vegetable based). The majority (65%) of foods identified could be classified as meats, grains, or fruits/vegetables. Fruits and vegetables comprised 42% (38/90) of all foods; meats 12% (11/90); and grains 11% (10/90). Sauces (6%, 5/90), sweets (4%, 4/90), and desserts (4%, 4/90) were also reported.

**Table 2:** Classification of foods reported (n = 90) by Senegalese men in the 24-hour recall interview

Meat/Poultry/Fish/Meat Alternatives (n = 11):	Grains (n = 10):	Fruits and Fruit Juices (n = 17):
Fish (Thiouf, Codfish, Yaboy)	Bread	*Fruits (n-12)*
Beef/Cow	Porridge	Mango
Sausage	Cereal paste/millet	Coconut
Chicken	Flour/baking ingredients^b^	Cola nut
Ox	Fataya (pound wheat)	Banana
Goat	White rice	Raisins
Sheep	Couscous	Papaya
Pork	Pita Bread	Pear
Eggs/Omelet^a^	Pasta/macaroni	Watermelon
Chickpeas	Spaghetti	Apple
Peanuts		Grapes
		Sapoti
		Maad bi
Milk/Dairy Products (n = 4):		*Fruit Juices (n = 5)*
Powdered mik		Quinquiliba juice
Liquid milk		Monkey bread juice
Cheese		Guava juice
Yogurt		Pineapple juice
		Orange juice
**Vegetables and Vegetable Juices (n = 21):**	**Sweets/Sweeteners (n = 4):**	**Desserts (n = 4):**
***Vegetables (n = 19)***	Sugar	Cake
Tomato	Honey	Chocolate croissant
Lettuce	Chocolate	Milk flavored biscuit
Carrot	Cocoa powder	Hard mint candy
Cabbage		
Corn	**Sauces (n = 5):**	
Eggplant	Bissap paste/sauce	**Beverages (not fruit/veg) (n = 7):**
Okra	(hibiscus)	Water
Garlic	Lemon sauce	Tea
Onion	Red pepper powder/sauce	Coffee
Potato	Peanut paste/sauce	Soda
Turnip	Sauce	Fanta
Cucumber		Beer
Green bean	**Fats (n = 3):**	Red wine
Green pepper	Butter	
Green pea	Mayonnaise	
Petit pois	Oil	
Broccoli		
Green olive	**Condiments/Spices (n = 4):**	
Cowpeas	Salt	
***Vegetable Juices (n = 2)***	Black pepper	
Root juice	Mustard	
Ginger juice	Ketchup	

^a^Excludes eggs found in cakes, breads, mayonnaise, pasta, etc.

^b^Includes flour, cornmeal and yeast disaggregated from recipes for items like pizza and fataya

Table 3 shows the most commonly consumed foods with "common" defined as ranking in the top 25% of all foods consumed in the category. For example, the top 3 meats out of a total of 12 meats are shown. The most commonly reported (median daily intake) meats were fish (1.17 oz/d), beef (2.61 oz/d), and ox (2.00 oz/d); milk/dairy product was cheese (0.38 oz/d); grains were bread (18.07 in^3^/d) and rice (0.75 cup/d); fruits were mango (1.00 whole fruit/d) and orange juice (1.25 cup/d); vegetables were potato (0.5 cup/d), carrot (0.33 cup/d), lettuce (0.65 cup/d) and tomato (0.06 cup/d); and beverages were water (5.09 cups/d), coffee (0.06 cups/d), and tea (0.56 cups/d). Other foods commonly reported were sugar (0.71 tbsp/d), sauce/peanut paste (4.43 tbsp/d), and butter (0.71 tbsp/d). Although consumption of tomato and carrot were commonly reported, they were consumed as a part of mixed dishes resulting in a low median daily intake. Bread was most commonly reported as baguette and is shown in the measure of inches cubed. Water was the only food item reported having been consumed by 100% of the men interviewed. Reporting of condiments, spices, and desserts was low overall. There were no statistically significant differences in consumption of each food by tribal affiliation, education, smoking status, urban/rural residence, BMI or age (data not shown).

**Table 3:** Daily intake (median and interquartile range (IQR)) of the most commonly consumed foods (top 25%) by Senegalese men (n = 50), by food category.

Food category	N (%)	Daily Intake (Median (IQR))
**Meats/Meat Alternatives (n = 11)**		
Fish (oz)	29 (58)	1.17 (0.33, 4.00)
Beef (oz)	18 (36)	2.61 (2.00, 3.00)
Ox (oz)	15 (30)	2.00 (2.00, 4.00)

**Milk/Dairy Products (n = 4)**		
Cheese (oz)	6 (12)	0.38 (0.17,0.50)

**Grains (n = 10)**		
Bread (in^3^)	45 (90)	18.07 (13.30, 26.60)
Rice (cup)	32 (64)	0.75 (0.50, 1.50)

**Fruits and Fruit Juices (n = 17)**		
Mango (#)	9 (18)	1.00 (1.00, 1.00)
Orange Juice (cup)	5 (10)	1.25 (1.00, 2.18)
Pineapple Juice (cup)	4 (8)	0.65 (0.30, 1.90)
Banana (#)	4 (8)	1.00 (0.75, 2.25)
Pear (#)	4 (8)	0.75 (0.50, 1.00)

**Vegetables and Vegetable Juices** **(n = 21)**		
Potato (cup)	18 (36)	0.50 (0.25, 0.75)
Carrot (cup)	16 (46)	0.33 (0.14, 1.27)
Lettuce (cup)	7 (14)	0.65 (0.22, 0.73)
Tomato (cup)	5 (10)	0.06 (0.06, 0.06)

**Sweets/Sweeteners (n = 4)**		
Sugar (tablespoon)	13 (26)	0.71 (0.53, 1.06)

**Sauces (n = 4)**		
Sauce/Peanut Paste (tablespoon)	31 (62)	4.43 (2.00, 11.08)

**Fats (n = 3)**		
Butter (tablespoon)	13 (26)	0.71 (0.71, 1.41)

**Condiments/Spices (n = 4)**		
Ketchup (tablespoon)	1(2)	2.00 (2.00, 2.00)
Mustard (ounces)	1(2)	1.00 (1.00, 1.00)

**Desserts (n = 4)**		
Milk Biscuit (pieces)	2(4)	0.64 (0.30, 1.00)
Candy (pieces)	2(4)	3.00 (1.00, 5.00)

**Beverages (not fruit/vegetable) (n = 7)**		
Water (cup)	50 (100)	5.09 (3.19, 6.13)
Coffee (cup)	29 (58)	0.06 (0.06, 0.09)
Tea (cup)	27 (54)	0.56 (0.19, 0.56)

Even though our study sample represents a relatively educated group, with 40% having at least some college education, we report that traditional foods are still commonly consumed in the Senegalese diet. Of the foods reported, the majority were fruits and vegetables. The World Health Organization's Global Strategy on Diet, Physical Activity and Health emphasizes the need to increase intake of fruits and vegetables while limiting refined sugars, saturated fat, and sodium [10]. In 2006, Holdsworth and colleagues reported that Senegalese women had reasonably good knowledge of how diet affects non-communicable diseases [11]. However, Holdsworth et al reported poor knowledge of the benefits of eating fruit and vegetables for the prevention of certain cancers. Although men were omitted from Holdsworth's study, our data suggest that despite a lack of health knowledge, Senegalese adults are meeting current WHO recommendation to consume at least 5 servings of fruits and vegetables. Each meal typically contained fruits and vegetables, either consumed alone or as a part of mixed dishes such as stews and soups. Many different types of grains were reported such as fataya, millet, and couscous but the most commonly reported grains (bread and rice) are foods that are also commonly consumed in the US/Western diet. The impact of these similarities requires further investigation. Snack products were not reported but sodas were. Beef and ox were commonly reported meats but the daily median portion size was much below US recommended serving sizes (i.e., 3-4 oz of meat/svg). The majority of foods reported in this study were primarily native to Senegal, and typical of the Senegalese diet.

A limitation of this work was the small sample size. Although we suspected intake would vary by demographic characteristics, it did not, and this may in part have been due to the low power to detect statistically significant differences in this sample set. We did not see differences when data were stratified by rural and urban residence. Also, most of the men in our sample had meals prepared by others and clarification by the meal preparer was sought when necessary. We were only able to conduct one 24 hr dietary recall interview. Multiple interviews or alternative measures of diet such as food frequency questionnaires were not feasible but could have given us more information about variability in dietary intake. Future studies may benefit from both a larger sample size and additional measures of food intake. The strengths of this study include the use of a trained interviewer to conduct the 24 hour dietary recall interview. We included an overall well-educated sample from a culture that values honesty and who were likely to have recalled their diets accurately. Additionally, the sample had variability in participant age with a range from 20 to 62 years.

The role of diet and nutrition in the prevention of chronic diseases such as cancer and cardiovascular disease in the Senegalese population is understudied. This work provides an important first step in characterizing the diet of Senegalese. While data from this study cannot be used to directly compare the Senegalese diet to the American diet, we see that characteristics of a Western diet are present in Senegal. In the US, Black populations reportedly have lower intake of fruits, vegetables, and whole grains, and higher intake of meats, and a less healthy diet overall than other racial/ethnic groups [12,13]. The Senegalese diet has more fruits and vegetables, and less meat than has been documented in the typical African American diet. Additional research is needed for more precise, standardized, dietary assessment, and to assess the variability in dietary intake for foods or food groups that are associated with chronic diseases. These efforts will further our understanding of the role of diet in the etiology and prevention of diet-related diseases.

## Competing interests

The authors declare that they have no competing interests.

## Authors' contributions

Substantial contributions to conception and design were made by CA, SB, TR, and CZJ. Substantial contributions to acquisition of data, or analysis and interpretation of data were made by CA, SB, MC, MF, MJ, SG, and ES. Involvement in drafting the manuscript or revising it critically for important intellectual content was made by all authors. All authors have given final approval of the version to be published.
